# Case report: A case of severe lower limb necrotizing fasciitis caused by an insulin injection has been reported

**DOI:** 10.3389/fmed.2024.1514241

**Published:** 2025-01-07

**Authors:** Yang Ma, Jinshuo Tang, Fei Wang, Enbo Liu, Jianlin Zuo

**Affiliations:** Department of Orthopedics, China-Japan Union Hospital of Jilin University, Changchun, China

**Keywords:** necrotizing fasciitis, *Escherichia coli*, finger separation test, vacuum sealing drainage, diabetes, insulin injection

## Abstract

Necrotizing fasciitis (NF) is a rare but life-threatening soft tissue infection, often accompanied by severe systemic toxicity. Early detection and prompt treatment are critical for survival. We report a case of NF in a 53-year-old diabetic woman following a subcutaneous insulin injection in the thigh. The patient presented with severe local pain as the initial symptom, and *Escherichia coli* was the sole isolated pathogen, which is seldom reported in the current literature. We combined with existing literature and clinical manifestations observed in NF patients at our hospital, offer valuable guidance for clinicians in recognizing and responding to NF.

## 1 Introduction

Necrotizing fasciitis (NF) is a rare but severe soft tissue infection characterized by progressive necrosis of subcutaneous tissue, particularly superficial and deep fascia. Although its incidence is low, NF has a high mortality rate of 20% to 30% (1). The condition is often accompanied by severe systemic toxicity and requires prompt diagnosis and aggressive treatment to prevent rapid fatal outcomes (2). Most NF patients have underlying conditions, such as immunosuppression, cancer, or diabetes, with diabetes being notably associated with NF (3), accelerating infection progression and worsening prognosis, ultimately increasing the risk of death.

**Figure 1:**
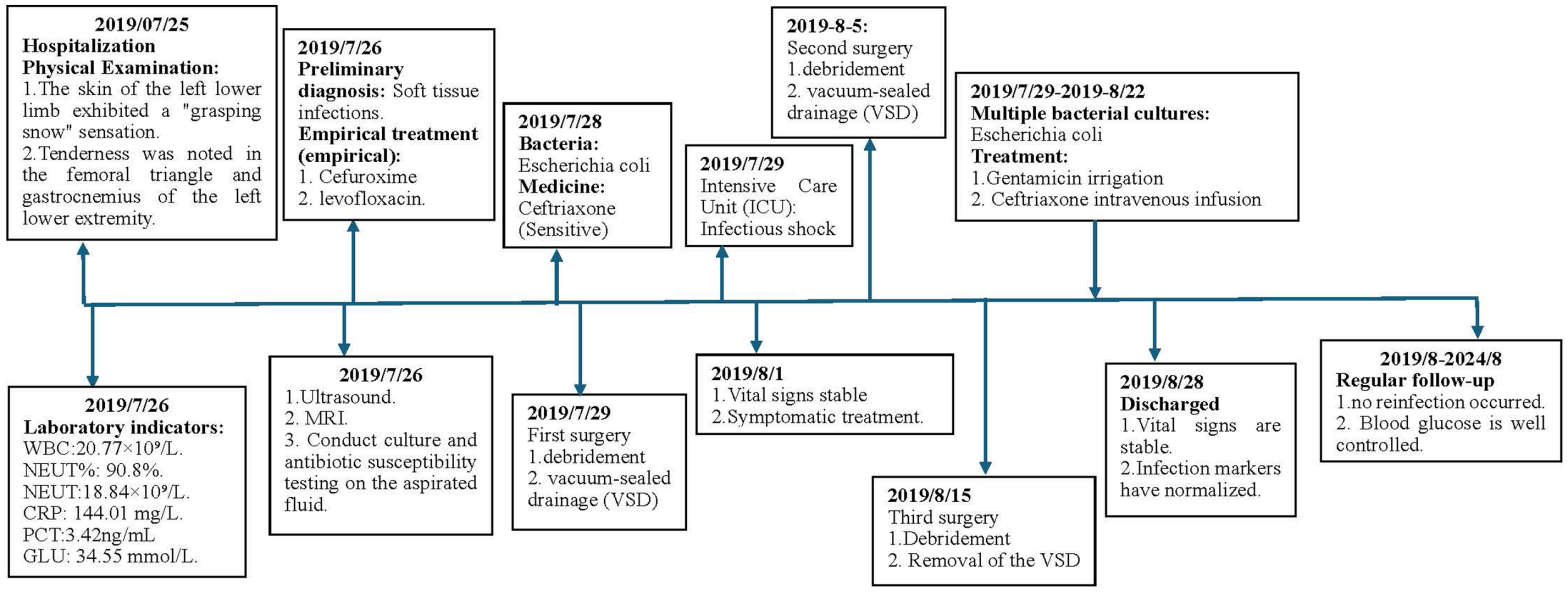
This image shows the timeline of the patient’s entire treatment process.

In most cases, necrotizing fasciitis (NF) is caused by the synergistic action of multiple microorganisms, though single-bacterium infections are occasionally observed. This report presents a case of NF in a patient with type 2 diabetes following an insulin injection in the thigh, with *Escherichia coli* identified as the sole pathogen. The case contributes to clinical data and serves as a reference for future practice. The timeline of the treatment process is shown in Figure 1.

## 2 Case report

A 53-year-old female patient was admitted to the hospital with pain and swelling in her left lower limb, which had persisted for five days. She has a 15-year history of poorly controlled type 2 diabetes mellitus, managed with insulin therapy.

**Figure 2:**
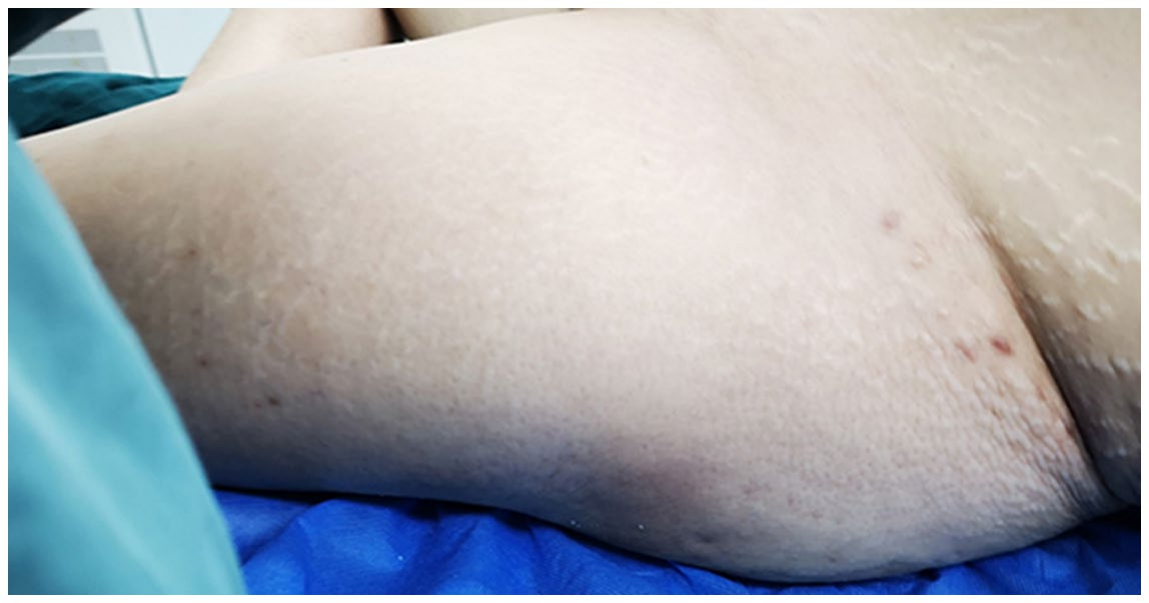
The skin on the left thigh exhibited mild redness, scattered blisters, and noticeable wrinkling.

Physical Examination: significant swelling in the left thigh and knee, with mild swelling of the left calf. The skin around the knee joint was red, while the left thigh exhibited slight redness, scattered blisters, and skin wrinkling (as illustrated in Figure 2). The skin temperature was elevated, and the left lower limb felt unusually cool to the touch. The skin of the left lower limb exhibited a “grasping snow” sensation. Tenderness was noted in the femoral triangle and gastrocnemius of the left lower extremity. Paresthesia was present, but the arterial pulse remained normal.

Laboratory indicators: white blood cell count: 20.77 × 10^9^/L, neutrophil percentage: 90.8%, neutrophil count: 18.84 × 10^9^/L, hemoglobin: 110 g/L, albumin: 22.07 g/L, c-reactive protein (CRP): 144.01 mg/L, procalcitonin: 3.42 ng/mL, blood glucose: 34.55 mmol/L.

**Figure 3:**
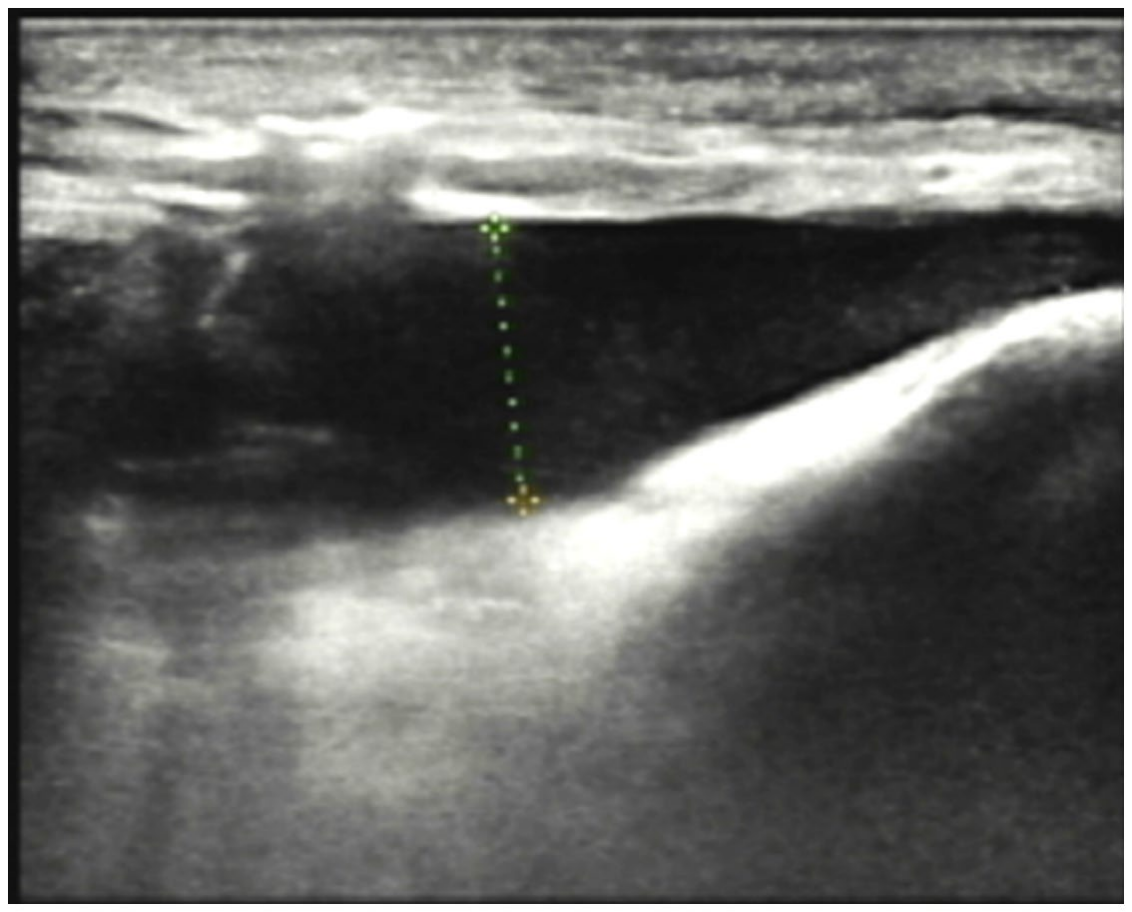
Ultrasound revealed subcutaneous soft tissue edema with a prominent dark fluid area.

Imaging Studies: Ultrasound revealed diffuse thickening of the subcutaneous soft tissue in the left lower limb, with enhanced and disordered echogenicity. Notably, there was a significant area of dark fluid in the left thigh and knee joint, as illustrated in Figure 3. Magnetic Resonance Imaging (MRI) of the left knee showed effusion in both the articular cavity and suprapatellar capsule, gas accumulation in the suprapatellar capsule, and surrounding soft tissue swelling. These findings suggested a possible infection with abscess formation.

**Figure 4:**
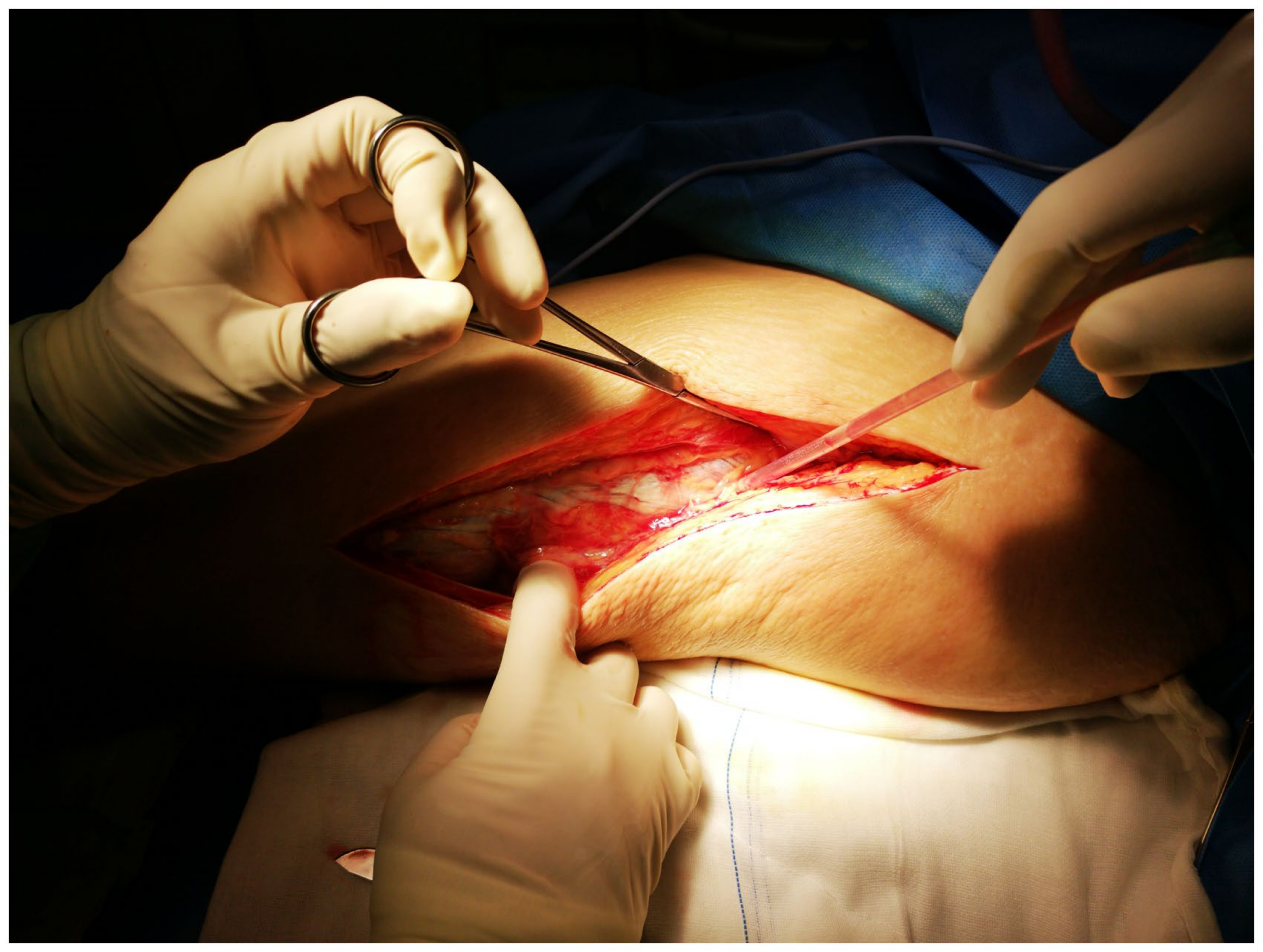
The finger test was positive, with easy separation of the skin from the fascia.

Process of treatment: Upon admission, the patient received empirical treatment with cefuroxime for infection, albumin infusion, and omeprazole to protect the gastric mucosa. A joint cavity puncture was performed to extract effusion for bacterial culture. However, the patient’s symptoms progressively worsened, with severe pain not conforming to the physical signs. Necrotizing fasciitis was suspected, leading to debridement of the left lower limb. During the operation, a substantial amount of cloudy brown pus was released from the subcutaneous soft tissue and muscle compartments of the left thigh and knee, along with significant necrotic tissue. The “finger separation test” yielded a positive result, as illustrated in Figure 4.

**Figure 5:**
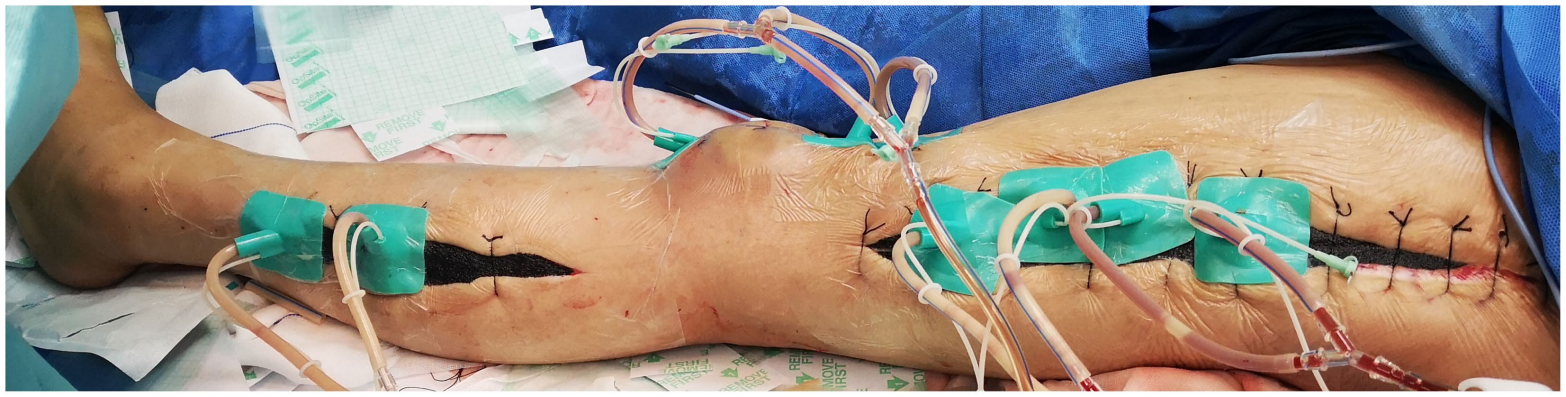
Debridement and vacuum sealing drainage (VSD) were performed.

While there was evident edema in the subcutaneous soft tissue of the left lower leg, no pus or abscess formation was observed in the subcutaneous or muscle compartments. Following repeated debridement, irrigation, and hemostasis, the wound was closed using Vacuum sealing drainage (VSD), as shown in Figure 5.

The patient exhibited cool, moist skin and drowsiness postoperatively, blood pressure:75/35 mmHg, Heart rate:120 beats/min, urine output<100 mL. white blood cell count:29.71 × 109/L, procalcitonin:12.51 ng/mL. The total blood loss during the operation was approximately 150 mL. The clinician assessed the patient as being in septic shock and transferred them to the ICU for endotracheal intubation. Ceftriaxone, a sensitive antibiotic, was administered for infection control, along with intravenous fluids and blood transfusions to correct the shock state, and insulin was given to stabilize blood glucose levels. By the third postoperative day, the patient’s vital signs had normalized, and the shock state was resolved, allowing for transfer back to the orthopedic ward. The patient underwent debridement surgery on the 19th and 28th days of his illness and made a significant recovery. He had a total hospital stay of 38 days. Upon discharge, his vital signs were stable, and infection indicators had returned to normal. The patient was followed up regularly for 5 years, during which no reinfection occurred. The patient is highly satisfied with the treatment process.

## 3 Discussion

The case reports a case of a diabetic patient who experienced difficulty tolerating insulin injections in the abdomen. Without medical guidance, the patient self-administered insulin injections in the thigh, neglecting to sterilize the injection site each time. During treatment, 14 cultures of pus were performed, and after excluding negative results and contamination, all cultures confirmed the presence of *Escherichia coli* as the sole pathogen, causing subacute necrotizing fasciitis. In this case, timely debridement was performed following the confirmation of the diagnosis. Multiple incisions were made based on the extent of involvement in the left lower limb. Vacuum sealing drainage (VSD) was employed to promptly remove pus and exudate from the cavity, reducing wound tension and accelerating healing. Based on drug sensitivity testing results, ceftriaxone was selected for antimicrobial treatment postoperatively.

According to existing literature, polymicrobial infections are more common than single-microbe infections in cases of NF (4, 5). Bacteria commonly causing necrotizing fasciitis include Group A Streptococcus (4), *Staphylococcus aureus* (5), and mycobacteria (6–10). Allison Perz et al. (11) reported that wound cultures from their patients tested positive for Mucor and methicillin-resistant *Staphylococcus aureus*. Shibayama et al. (9) reported a case of necrotizing fasciitis caused by *Mycobacterium abscessus* infection. Regev et al. (12) reported two cases of necrotizing fasciitis caused by *Staphylococcus aureus*. Reports of necrotizing fasciitis caused by *Escherichia coli* are rare, especially when *Escherichia coli* is the sole pathogen. Among 83 necrotizing fasciitis cases reported by Brook and Frazier (13), only six involved a single pathogen: four caused by Group A Streptococcus and two by *Staphylococcus aureus*, with no cases of *Escherichia coli* as the sole pathogen. Among 51 necrotizing fasciitis cases reported by McHenry et al. (14).19 involved a single pathogen. Group A Streptococcus was the most common, identified in 10 cases, followed by *Staphylococcus aureus* in two cases, and *Escherichia coli* in just one case. The diabetic patient reported in this study developed subacute necrotizing fasciitis caused by *Escherichia coli* as the sole pathogen, a rare occurrence. Local inoculation of *Escherichia coli* by subcutaneous insulin injection was the likely portal of entry in this patient.

Currently, clinical diagnosis of necrotizing fasciitis (NF) faces numerous challenges. As illustrated in our case, the early clinical signs of NF are often nonspecific, with significant manifestations including ecchymosis, bullae, and skin wrinkling in only a few instances. Ultrasound and MRI can serve as valuable adjunctive diagnostic tools for NF. Kwee’s study indicated that MRI, exhibiting T2-weighted hyperintensity of the deep fascia, demonstrates high sensitivity and moderate specificity for diagnosing necrotizing fasciitis (15). Sartelli’s study indicates that ultrasound imaging in NF shows a loss of normal tissue architecture, characterized by a “cobblestone” appearance. Additionally, there is an irregular thickening of the fascia and abnormal fluid accumulation along the fascia, which appears as low echogenicity (16).

Due to the similarity between the common clinical manifestations of NF and non-necrotizing infections such as cellulitis, especially in the early stages with nonspecific symptoms like swelling and pain (3), NF is often misdiagnosed. Additionally, the initial skin lesions in NF may appear benign, and hemodynamics can remain stable. Therefore, necrotizing fasciitis must be clinically differentiated from cellulitis, erysipelas, and abscesses, which are common types of soft tissue infections. Cellulitis is characterized by widespread, diffuse inflammation of the skin and subcutaneous tissue, presenting with localized redness, warmth, swelling, pain, and poorly defined borders. The central area is typically the most pronounced, with gradual spread to surrounding tissues. Compared to NF, cellulitis is less painful and generally lacks skin numbness. Laboratory findings often reveal elevated white blood cell counts and neutrophil proportions, but inflammatory markers increase less significantly than in NF. Blood cultures are typically negative unless the infection has progressed to sepsis. Ultrasound shows thickened subcutaneous tissues with diffuse hyperechoic changes. MRI may reveal diffuse subcutaneous edema with high T2-weighted signal intensity but no extensive necrosis or fascial destruction. Erysipelas, commonly caused by group B streptococcus, primarily affects the lower extremities and face. It is characterized by sharply demarcated, bright red patches of skin that are slightly raised and blanch under pressure, occasionally accompanied by blisters. Patients may experience systemic symptoms such as chills and fever; however, pain and swelling are generally milder compared to necrotizing fasciitis, and skin necrosis is uncommon. Laboratory findings often show elevated white blood cell counts, predominantly neutrophils, with a possible increase in antistreptolysin O(ASO) titers. Abscesses are clinically characterized by localized pain, redness, and swelling, with fluctuation on palpation, indicating pus formation. The inflammatory response is typically confined to the surrounding tissues, distinguishing it from the rapid spread seen in necrotizing fasciitis (NF). Laboratory tests often show elevated white blood cell counts and neutrophil proportions. Blood cultures are usually negative, while pus culture can identify the causative pathogen. Tutino et al. (17) identified key factors for distinguishing cellulitis from NF, including recent surgery, disproportionate pain relative to clinical findings, hypotension, skin necrosis, and hemorrhagic blisters. Teelucksingh et al. (18) found that exacerbating pain through specific muscle activity helps differentiate muscle and fascial involvement in cellulitis and NF. This technique is considered a useful diagnostic clue and an effective method for distinguishing superficial infections. Additionally, in atypical cases, the involvement of dermatologists helps differentiate necrotizing fasciitis from rare similar conditions, such as necrotizing pyoderma.

We have summarized the following clinical signs from the reported literature that warrant suspicion of NF:1. Sudden onset of systemic toxic reactions post-surgery. 2. Severe pain symptoms that do not align with the physical examination findings. 3. Extensive skin necrosis, accompanied by palpable crepitus, abnormal sensations, or even loss of limb function. 4.No significant improvement after antibiotic treatment, with continued disease progression. For cases where necrotizing fasciitis (NF) cannot be definitively diagnosed, guidelines and research recommend surgical exploration to confirm the diagnosis and prevent treatment delays. Surgical exploration is regarded as the gold standard for diagnosing NF.

At present, Surgical intervention remains the primary treatment for necrotizing fasciitis (NF), with drug therapy playing a supportive role. The goal of debridement is to remove infected and necrotic tissue, thereby preventing further spread through the fascia and bloodstream. Latifi (19) found that early, timely surgical intervention reduces mortality, lowers the risk of septic shock, decreases the number of debridements required, and shortens hospital stays. Antimicrobial therapy is an indispensable adjunct in the treatment of NF, effectively eliminating postoperative residual bacteria and inhibiting their growth. Before the pathogen is identified, broad-spectrum antibiotics such as vancomycin can be administered empirically. Once the pathogen is confirmed through sensitivity testing, targeted antibiotics should be used for more precise treatment.

## 4 Conclusion

In conclusion, for high-risk groups prone to NF, such as individuals with diabetes, immunocompromised conditions, the elderly, and those with obesity, it is crucial to maintain proper skin hygiene and avoid injury, particularly for those with underlying conditions. Actively manage conditions like diabetes and immunosuppression by controlling blood sugar levels and enhancing immunity. Avoid exposure to contaminated environments and promptly clean and disinfect any injuries. For diabetic patients, strict aseptic techniques must be followed during insulin injections: ensure a clean environment, disinfect the injection site with an alcohol swab, allow it to dry, perform the injection properly, and dispose of needles in a designated sharps container to prevent infection. The early clinical signs of NF are often non-specific, making early recognition and diagnosis critical. Prompt debridement and drainage, possibly requiring multiple surgeries, are essential for treatment. Concurrently, antibiotic therapy should begin with broad-spectrum antibiotics, which can be adjusted to targeted antibiotics once the causative bacteria and susceptibility results are confirmed. Throughout antibiotic treatment, adequate dosing and duration are crucial. Additionally, managing underlying conditions and providing nutritional support are vital to improving outcomes.

## Data Availability

The raw data supporting the conclusions of this article will be made available by the authors, without undue reservation.
